# Effects of landscape simplicity on crop yield: A reanalysis of a global database

**DOI:** 10.1371/journal.pone.0289799

**Published:** 2023-12-14

**Authors:** Michael Biwalib Madin, Katherine S. Nelson

**Affiliations:** Department of Geography and Geospatial Sciences, Kansas State University, Manhattan, Kansas, United States of America; University of Ferrara, ITALY

## Abstract

Ecological theory on diversity suggests that agriculture requires sufficient biodiversity, ecological function, and critical ecosystem services to remain sustainable and resilient. As such, research related to the effect of ecosystem services and diversity on crop yields has increased significantly in the past decade. One such study by Dainese and colleagues that presented a global synthesis of a compiled database of 1,475 crop experiments related to pollination and pest control ecosystem services and crop yields quickly garnered attention in the literature with more than 540 citations since its publication in 2019. Given the strong influence of this study on the research on diversity and agricultural production, we conduct a reanalysis on the publicly available dataset from the global synthesis study to test the robustness of findings to modeling approach and assumptions. In our reanalysis we apply ordinary least squares regression methods rather than Bayesian path analysis to the same data to examine the robustness of observed field-scale landscape diversity-ecosystem services-crop yield relationships. The result of our reanalysis supports the findings of Dainese and colleagues, illustrating the robustness of findings that suggest that increasing landscape simplicity is associated with lower rates of pollination and pest control ecosystem service provisioning and lower crop yields. However, our analyses also suggest that provisioning of pollination and pest control services account for only a small fraction of the total effect of landscape simplicity on crop yields. Furthermore, we find that management and soil health may mediate the effects of landscape simplicity on ecosystem services and crop yields. While our results complement previous findings for landscape simplicity and ecosystem services, they also indicate that above and below ground ecosystem services are not mutually exclusive but concurrently contribute to support crop production in agriculture.

## Introduction

Global biodiversity loss and environmental change pose significant challenges to sustainable agriculture production and global food security [1]. Ecological theory on diversity suggests that agriculture requires sufficient biodiversity, ecological function, and critical ecosystem services to remain sustainable and resilient [2–4]. Crop yields are one essential component of the benefits offered by agricultural landscapes and frequently serve as an indicator for assessment of sustainable agricultural production [5, 6]. As such, several global meta-analyses have examined the effect of ecosystem services and diversity on crop yields [6, 7]. These studies suggest that reduction in diversity via landscape simplification affects ecosystem services by reducing both richness and abundance of service-providing organisms, such as pollinators and natural predators, with negative consequences for crop yields.

Understanding the role of diversity in the functioning of ecosystems has important implications for agriculture. Prior research has shown that diversifying crops and agricultural landscapes leads to increases in yield relative to monocultural simplified landscapes [4, 8]. Theory holds that diversity promotes stability and resilience in agriculture production systems in part by enhancing ecosystem services provision [2]. Diversity promotes, for example, pollination of crops, biological pest control, maintenance of soil structure and fertility, reduced erosion, nutrient cycling, and soil moisture retention and distribution [3, 9]. At the field-scale, evidence shows that higher diversity of habitat surrounding crop fields results in more natural enemies, fewer pests, and in some cases, a trend toward greater crop yield [10]. Relatedly, increases in soil fauna diversity have been demonstrated to increase crop productivity by 35% across ecosystems [11]. Results at a broader landscape scale indicate that crop production is more responsive to the number of distinct crop types cultivated on a landscape than their cultivated extent, and that increasing diversity in agricultural systems that are already diverse brings the highest yield gains [12, 13].

While simplification of agricultural landscapes has frequently been shown to reduce ecosystem health and trade long-term agricultural sustainability for short-term yield gains [7, 14, 15], understanding of how landscape simplification or landscape simplicity impacts crop yields remains debatable. Mixed and contradictory findings are apparent in the literature, with factors such as biophysical conditions, regional socio-economic context, on-farm management practices, diversity metric, scale, and modeling technique frequently identified as potential reasons behind these differences [16, 17].

Biophysical conditions and on-farm management practice, in particular, have been shown to be strongly associated with agricultural landscape diversity and crop productivity [18–20]. These factors may both directly affect crop yields and ecosystem services and may moderate the relationships between landscape diversity, ecosystem services, and crop yields. For example, studies by Karp et al. [16] and Seufert et al. [6] showed that the potential positive benefits of more diverse agricultural landscapes, along with other ecologically friendly management practices, are such that yields from diverse organic systems can almost match conventional farms for certain crop types, particularly fruit crops. Well managed organic and diverse systems have also been shown to reduce agriculture impacts on the environment, leading to improvement in the use of natural resources to preserve the environment and ensure sustainable production [21, 22] and to support higher levels of biodiversity, hosting an average of 30% more species compared to conventional farmland [23]. This abundance of species plays a crucial role in supporting various ecosystem services, including pollination, nutrient recycling, and the provision of clean water and air. Despite the importance of climate or weather variability and soil characteristics for crop yields [23–25], and evidence of climate-related differences in diversity-yield relationships [24], these factors are not consistently considered in studies evaluating how diversity is related to field-scale ecosystem services or crop yields around the world [25–30].

Unfortunately, while some notable datasets have been published, reanalysis of this data to assess sensitivity of findings to modeling approach and assumptions is lacking in the published literature [31–33]. To address these research gaps, we conduct a reanalysis of a compiled global database published by Dainese et al [7]. We conduct modeling to examine the robustness of observed field-scale landscape diversity-ecosystem services-crop yield relationships to changes in the modeling approach. Specifically, we address the following questions.

1. How sensitive are findings about the relationships between landscape simplicity/diversity and ecosystem services and crop yields to modeling technique?
2. How sensitive are findings about the relationships between landscape simplicity/diversity and ecosystem services and crop yields to inclusion or exclusion of contextual climate, soil, and management factors?

Because Dainese et al. [7] used a Bayesian modeling technique, which for small samples sizes may be strongly influenced by assumed prior normal distributions, we first examine how the use of an ordinary least squares (OLS) modeling technique may alter findings. Similarly, while Dainese et al. [7] apply a form of path analysis to simultaneously examine the effects of landscape simplicity on ecosystem services, and through ecosystem services the effects on crop yields, we employ a simple series of OLS models to examine the total effect of landscape simplicity on crop yields, the effects of ecosystem services on yields, and the effects of landscape simplicity on ecosystem services. Second, we examine how explicit addition of previously excluded weather, soil, and management variables impact model results.

## Data and methods

### Dataset compilation

**Fig 1:**
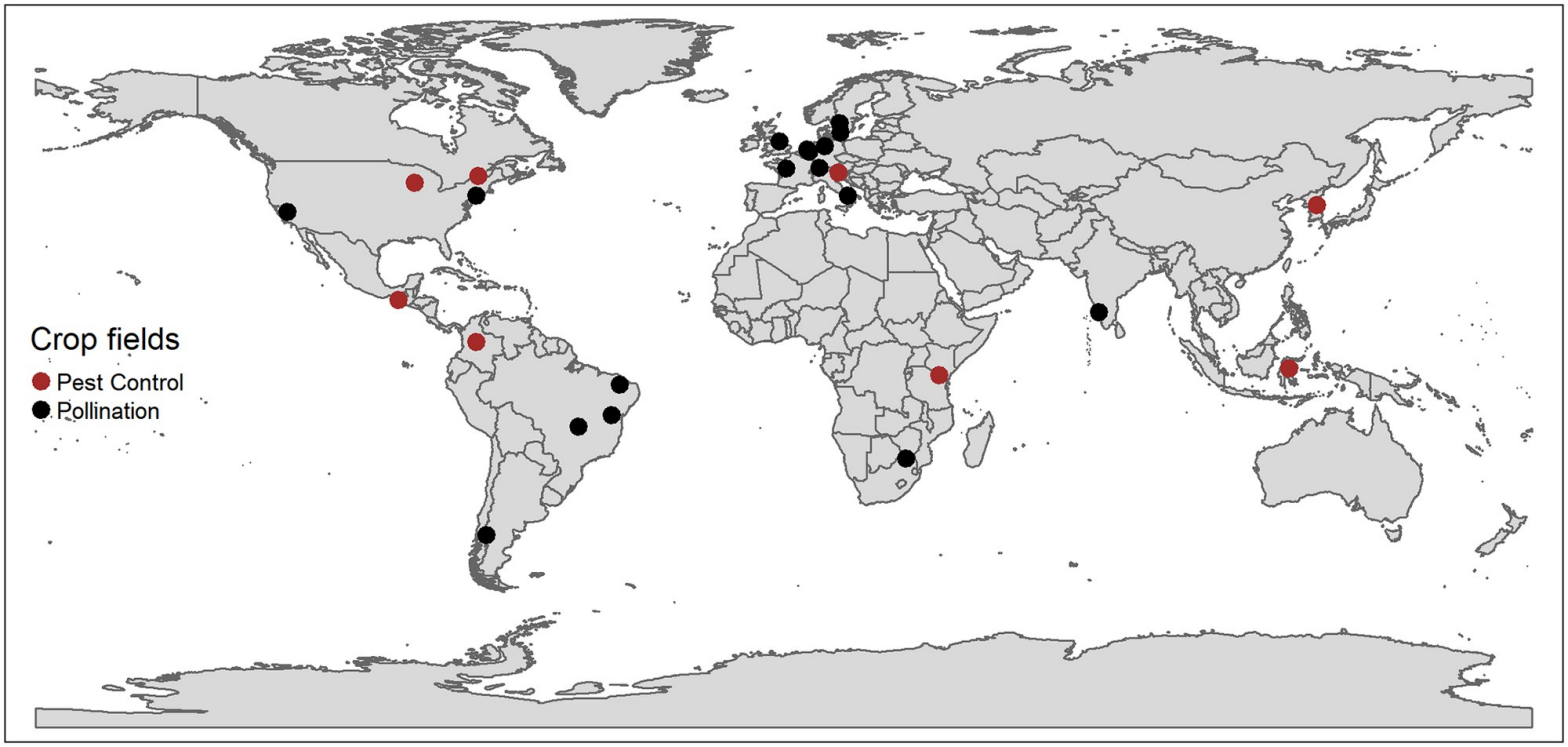
Distribution of reanalyzed crop field data. (The geographic boundaries data used in this study were obtained from R software Open-Source Data shapefiles found in the ‘arsenal’ package).

To compile a dataset for this study, we first obtained a publicly available global database on biodiversity and crop production [7]. The database contains information on 89 studies in 1,475 crop fields across 27 countries and 29 different crops. Given our focus on assessing relationships with crop yields, only “dataset3” and “dataset4” met our criteria and were included for reanalysis. The “dataset3” contains measures of pollination services and crop production while “dataset4” contains measures of natural enemy pest control and crop production. The datasets were merged, and duplicate experiments were removed. The merged dataset retained 40 studies, 581 crop fields, 18 countries, 24 crops, and 25 regions/cities (hereafter regions) of experiment locations across the world. See Fig 1.

Measures from the published Dainese et al. [7] dataset used in our reanalysis include PROD, LAND, POLL_IND, ES_PestControl, and Manag. PROD, henceforth *Yield*, is a crop production metric. Depending on the crop type, the authors [7] used either area-based yield or within plant yield (total number or mass of seeds or fruits per plant) which they then standardized (using z-scores) to put all the yield values on equivalent terms. *LAND* is a metric of landscape composition, or landscape simplicity, describing the percent of land use classified as a cropland within a 1 km radius around the center of each field. The percentage of cropland near fields or its inverse, percentage of natural cover, has been widely used as a proxy for characterizing landscape simplicity. This simple measure has been found to be related to biodiversity and above ground ecosystem services (such as pollination and natural pest control) in agriculture landscapes [7, 10, 16, 17, 34, 35]. It is also found to be often strongly correlated with other landscape metrics of compositional and configurational complexity [15, 17, 34, 36]. POLL_IND, henceforth *Pollination*, is a metric of pollination services that was measured by using differences in fruit set, average number of seeds per fruits, or fruit weight between plants with and without insect pollination. This was then converted into a pollination index using z-scores to put all values on equivalent terms. The ES_PestControl, henceforth *Pest Control*, metric was calculated in Dainese et al. [7] as the fraction or amount of each crop consumed, infested, or damaged by pests, where cages were used to exclude natural enemies to quantify differences in pest abundance or crop damage between plants with and without natural enemies. The standardized values of pest activity were inverted by multiplying by −1, as low values indicate positive contributions to the ecosystem service. Manag, henceforth *Management*, describes farm management types at the experiment sites which include monoculture of crop/grasses (control condition), conventional, organic, pond with semi natural habitat, and semi natural habitat lacking a pond.

Geographic coordinates of the locations for the 25 regions reported as experiment locations in Dainese et al. [7] were obtained from Google Earth^™^ and used to extract climate and soil information. Monthly minimum temperature (°C), monthly maximum temperature (°C) and total monthly precipitation (mm) were obtained from WorldClim for each study year [37, 38]. The WorldClim data was used to construct annual mean precipitation (*Precipitation*) and annual maximum temperature (*Max Temp*), metrics for each experiment region and year. These metrics were constructed by first calculating the sum of monthly precipitation in a year, maximum of monthly maximum temperature in a year, and minimum of monthly minimum temperature in a year for each pixel. Then the average across all pixels within a radius of the center of the region was calculated for each of these annual raster layers, where the radius was defined as one-half the square root of the region area. Soil texture class (*Soil*) was obtained from the World Harmonized Soil database [39] and was aggregated for crop experiment locations by taking the mode across all pixels within a radius of the center of the region to create the *Soil* metric (see the S1 Table for a detailed description of these calculations). All variables were standardized using z-scores, within crop type, prior to modeling to account for variations in physiological temperature, water, and soil condition requirements across crops.

### Modeling approaches

We structure our initial models following the same general model specifications as used in Dainese et al. [7], however we employ standard OLS regression as opposed to a Bayesian multivariate response (path analysis) modeling approach. The use of a series of simple OLS models reduces the chances of model overfitting and avoids the introduction of bias from selection of priors that Bayesian analysis on small sample sizes is prone to [40]. Therefore, our initial analyses examine a series of models specified as

Yield~LAND (1a)

Yield~Pollination (1b)

Yield~PestControl (1c)

Pollination~LAND (1d)

PestControl~LAND (1e)

Where (1a) assesses the association of landscape simplicity with crop yields, (1b) assesses the relationship between pollination and crop yields, and (1c) examines the relationship between pest control and crop yields. Models 1d) and 1e) examine the relationship between landscape simplicity and pollination and pest control where pollination and pest control ecosystem services are presumed to be dominant mechanisms through which landscape structure influences crop yields. Note that while Dainese et al. [7] did not include a direct effect of landscape simplicity on crop yields in their analysis, we do so to investigate the relative contribution of pollination and pest control services to the net effect of landscape simplicity on crop yields.

To examine the extent to which management and contextual climate and soil factors influence model findings, we add climate and soil variables and interactions between management and landscape simplicity and pollination and pest control to our model specification as in:

Yield~LAND*Manag+MaxTemp+Precipitation+Soil (2a)

Yield~LAND*Soil+MaxTemp+Precipitation+Manag (2b)

Yield~Pollination*Manag+MaxTemp+Precipitation+Soil (2c)

Yield~PestControl*Manag+MaxTemp+Precipitation+Soil (2d)

Pollination~LAND*Manag+MaxTemp+Precipitation+Soil (2e)

PestControl~LAND*Manag+MaxTemp+Precipitation+Soil (2f)

Model (2a) assesses the interactive relationship between landscape simplicity, management, and crop yield in addition to explicitly accounting for weather and soil conditions. We add *Management* as an interaction term because farm management practices, such as the use of fertilizers, pesticides, and irrigation, can directly impact yields and may also modify nutrient levels in plant tissue that impact the susceptibility of crop yield to environmental and pest-related plant damages as well as by directly impacting insect populations that provide yield-supporting ecosystem services across landscapes [41, 42]. In Model (2b), an interaction between landscape simplicity and *Soil* is examined as crops grown in soil conditions more optimal for crop production are likely to have nutrient levels that reduce their susceptibility to environmental and pest-related plant damages [41, 43, 44] and therefore, are likely to experience smaller relative benefits from decreasing landscape simplicity, while locations with poor soil quality may have plants more susceptible to pests and may see higher benefits from marginal increases in ecosystems services associated with landscape diversity [29, 45].

Model (2c) examines the interactive relationship between pollination services, management, and crop yields, while also considering weather and soil texture type. Here management is interacted with *Pollination* because fields that are diversified and managed organically, as well as landscapes that have a greater number of high-quality habitats, are known to have higher levels of abundance and richness of pollinators, such as bees [46]. Similarly, Model (2d) determines the synergistic association between *Pest Control*, management, and crop yield in addition to accounting for weather and soil texture types. Model (2e) assesses the interactive effect of landscape simplicity and management on pollination services while accounting for weather and soil texture. We interact landscape simplicity with management because the persistence of pollinators depends on both maintaining high-quality habitats around farms and implementing local management practices that do not directly harm pollinators [25]. Model (2f) examines the interactive effects between landscape simplicity and management on natural pest control while controlling for weather and soil texture. The interaction between landscape simplicity and management considers that natural pest control effects could be masked by management practices such as insecticide use while diversified landscapes act as reservoirs for natural enemies that have the potential to control insect pests [14].

There were 581 complete observations for models without pollination and pest control services (Models 1a, 2a, and 2b). The models with pollination variables have 395 complete observations, while those with pest control had a total of 186 observations. Missing values were excluded from our models’ analyses. The OLS models were run in R using the package lm.

## Results

### Part 1: Alternate linear modeling framework–series of linear equations using OLS

No significant direct relationship between landscape simplicity and crop yields is observed in our models. However, more diverse landscapes are associated with higher pollination and higher pollination is associated with higher crop yields (see Table 1). The OLS models do not indicate a significant relationship between landscape simplicity and natural pest control or between natural pest control and crop yields. In comparison, Dainese et al. [7] also found that increased pollination is associated with higher yields. However, they also found that increased natural pest control is associated with higher yields. (It should be noted that Dainese et al. [7] report values for natural pest control effects on yields for models using only insecticide-free experiment data whereas we use all data available). Relatedly, while we find a significant relationship between landscape simplicity and pollination services, Dainese et al. [7] find a relationship that is not significant at a 90% or higher confidence level.

**Table 1:** Linear models of landscape simplicity, pollination services, and predator activity on crop yields. The dependent variable in each model is shown in square brackets following the model name.

Model [Dependent Variable]	Predictor Variable	Estimated Coeff (Std Error)	AIC	Effect from Dainese et al. (Location where values are reported.)
*Model 1a [Yield]*	Land	0.0022 (0.0410)	1618.227	NA
*Model 1b [Yield]*	Pollination	0.2816 (0.0483)***	1072.395	0.344‡ (Fig 4A & S8 Table)
*Model 1c [Yield]*	PestControl	0.1191 (0.0729)	516.3204	0.148† (Fig 4B & S8 Table)
*Model 1d [Pollination]*	Land	-0.1552 (0.0496)**	1097.325	-0.057 (Fig 3A & S5 Table)
*Model 1e [PestControl]*	Land	-0.0224(0.0721)	520.6136	-0.028 (Fig 3B & S5 Table)

Significance level:

* p<0.10

** p<0.05

*** p<0.01.

Highest density intervals (HDIs), or credibility interval, from *Dainese et al*. [7] are represented as †90%, and ‡95%

### Part 2: Adding climate, soil, and management to the models

**Fig 2:**
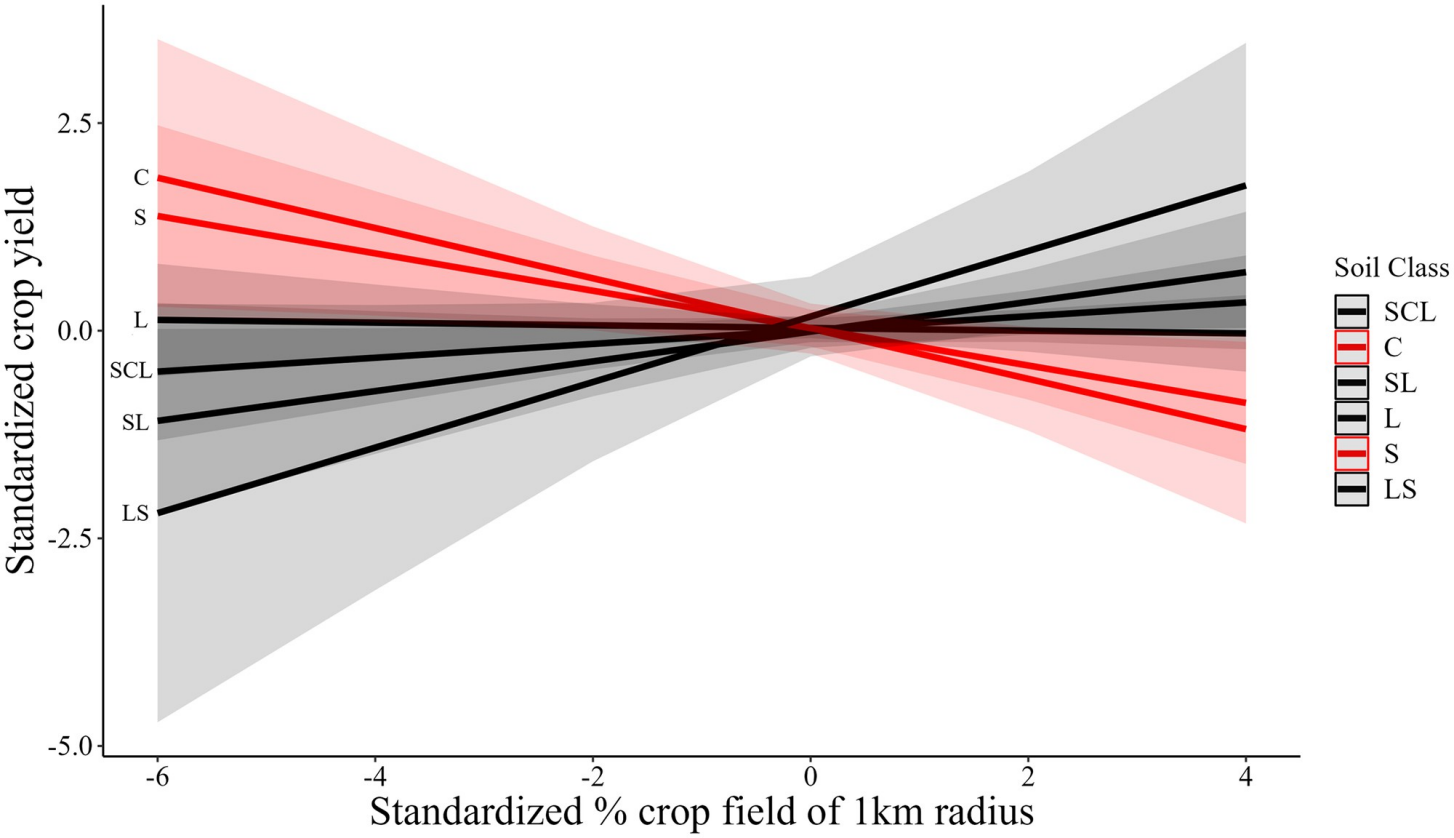
Model 2b predicted values of crop yields at given landscape simplicity in different soil texture classes. **Note:** SCL = Sandy-Clay-Loam, C = Clay, SL = Sandy-Loam, L = Loam, S = Sand, LS = Loamy-Sand. Solid lines represent the mean effect, and the colored bands represent a 90% confidence interval.

Models that explicitly account for regional climate, soils, and management indicate that landscape simplicity is associated with variable, and signficant, crop yield responses across different soil classes (Table 2, Model 2b) but not across management types. Specifically, higher levels of landscape simplificity (or lower levels of landscape diversity) are, on average, associated with reduced yields. However, higher levels of landscape simplificity are associated with higher crop yields in loamy and sand-clay-loam soil types, while in clay and sand dominated soils, higher landscape simplicity (lower landscape diversity) is associated with reduced crop yields (Fig 2). Thus, more fertile soils are associated with higher yields even in simplified landscapes while poorer soils are associated with higher yields in more diversified landscapes. While soil type had significant indirect effects on crop yields in Model 2b, soil effects were not significant in Model 2a. Similarly, climate variability and management practices were not found to be significantly associated with crop yield in either Model 2a or 2b.

**Table 2:** Estimated effects of landscape simplicity, ecosystem services, climate, and soil variables on crop yields. The dependent variable in each model is shown in square brackets following the model name.

Predictor Variables	Estimated Coefficient (Standard Error)					
	*Model 2a [Yield]*	*Model 2b [Yield]*	*Model 2c [Yield]*	*Model 2d [Yield]*	*Model 2e [Pollination]*	*Model 2f [Predator Activity]*
Land	-0.4388(0.4985)	-0.3027(0.1661)*	-	-	0.1719(0.4957)	0.0340(0.0788)
Pollination	-	-	0.2874(0.0597)***	-	-	-
PestControl	-	-	-	0.1600(0.0789)**	-	-
Manag [conventional]	0.3717(0.3749)	0.3988(0.3694)	-	-	0.8406(0.3833)**	
Manag [organic]	0.2180(0.3822)	0.2853(0.3770)	-0.1427(0.1170)	-0.1711(0.2202)	0.8396(0.3925)**	-0.3108(0.2243)
Manag [pond + semi natural habitat]	0.6262(0.4680)	0.5632(0.4605)	-	-	1.2979(0.4653)***	-
Manag [semi natural habitat lacking a pond]	0.4322(0.4827)	0.4185(0.4707)	-	-	1.1299(0.4799)**	-
Temp	-0.0223(0.0537)	-0.0250(0.0533)	-0.0070(0.1024)	-0.0576(0.0817)	-0.0030(0.1055)	-0.0412(0.0816)
Precip	0.0049(0.0490)	-0.0002(0.0486)	0.0392(0.0682)	0.0006(0.1142)	-0.0252(0.0726)	0.0295(0.1141)
Sand	0.0051(0.2419)	0.0054(0.2400)	0.0967(0.3511)	0.0708(0.3838)	-0.0540(0.3815)	0.5693(0.3843)
Loamy Sand	0.1929(0.3358)	0.1454(0.3337)	0.2339(0.3824)		-0.0480(0.3958)	
Loam	0.0298(0.2077)	0.0071(0.2062)	0.0816(0.2943)	-0.0358(0.3175)	-0.0451(0.3039)	-0.0168(0.3170)
Sandy loam	-0.0369(0.2175)	-0.0369(0.2175)	0.0891(0.3029)	-0.5888(0.4100)	-0.0150(0.3130)	-0.1043(0.3648)
Sandy clay loam	-0.0037(0.2012)	-0.0178(0.1996)	0.0783(0.2755)	-0.1826(0.3268)	-0.0530(0.2851)	0.1923(0.3262)
Land× conventional	0.4132(0.5009)	-	-	-	-0.3625(0.4996)	-
Land× organic	0.5172(0.5058)	-	-	-	-0.2826(0.5046)	-0.4760(0.2145)**
Land× pond + semi natural habitat	0.5917(0.5516)	-	-	-	-0.1958(0.5485)	
Land× semi natural habitat lacking a pond	0.0208(0.6858)	-	-	-	-0.0683(0.6819)	
Land× Loam	-	0.2865(0.1792)	-	-	-	-
Land× Loam sand	-	0.6973(0.3002)**	-	-	-	-
Land× Sand	-	0.0777(0.1978)	-	-	-	-
Land× Sandy clay loam	-	0.3861(0.1855)**	-	-	-	-
Land× Sandy loam	-	0.4818(0.1985)**	-	-	-	-
Pollination×Organic	-	-	-0.0073(0.1046)	-	-	-
PestControl×Organic	-	-	-	-0.3179(0.2166)	-	-
**AIC**	1640.3630	1623.2250	1088.3180	527.9804	1113.5590	528.0323

Significance level:

* p<0.10

** p<0.05

*** p<0.01.

***Note***: the reference category for soil texture class is clay and the management practices is control monoculture except for model 2c, 2d, and 2f where it is conventional.

Notably, the total effect of landscape simplicity on yields that we estimate is far larger in magnitude than the total effect suggested by Dainese and colleague’s path analysis, which can be estimated as the product of the significant indirect effects of landscape simplicity on yield [47]. Per their analysis, the total effect of landscape simplification on production through an effect on pollinator richness and pollination services (See Fig 4 of Dainese et al. [7]) would be -0.011 (-0.171*0.183*0.344) while our estimated effect of landscape simplicity on yields after accounting for soil type in Model 2b is -0.303.

The results for Model 2c are consistent with Model 1b, indicating that increased pollination is significantly associated with higher crop yield even after accounting for weather, soil, and management (Table 2, Model 2c). There were also no observed significant effects of soil and climate variables on crop yield in Model 2c. While no significant interactive effect between pollination and management on crop yields was observed, management type has a significant direct effect on pollination in Model 2e. Specifically, in comparison to monoculture crop control sites, both conventionally and organically managed sites are associated with similar levels of increased pollination. Sites in which management included semi-natural habitat had significantly higher pollination than the control, the conventional, and organic management sites. These results contrast with the results of Model 1d which suggest that increased pollination is associated with less crop-dominated landscapes, and with the results of Dainese et al. [7] that suggest that increases in the proportion of a landscape dedicated to cropping is associated with reduced pollination services (at a 80% credibility interval).

**Fig 3:**
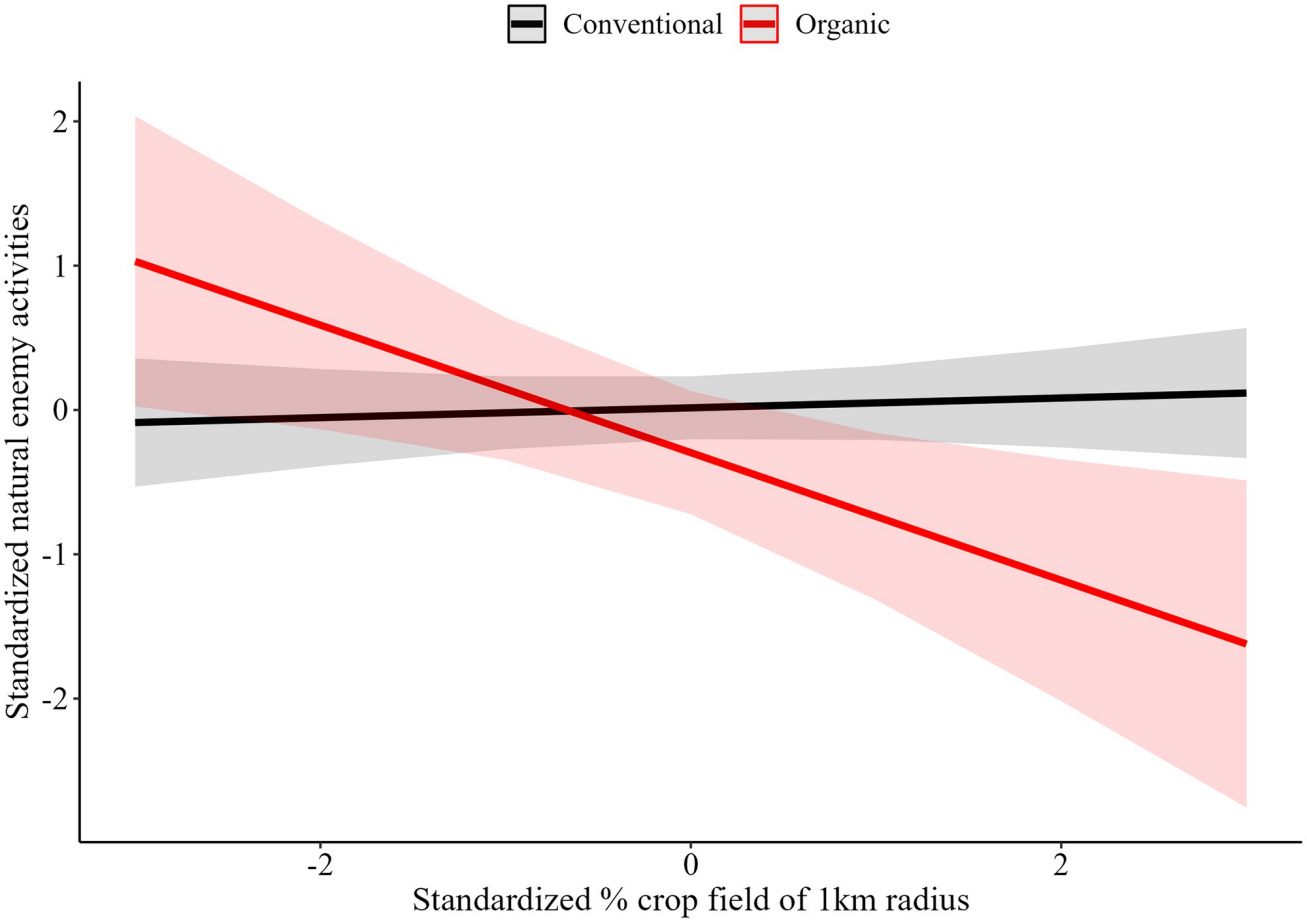
Model 2f predicted values of natural pest control at given landscape simplicity under different management practices. **Note:** Solid lines represent the mean effect, and the colored bands represent a 90% confidence interval.

Natural pest control alone did not have a significant direct effect on crop yields as shown in Model 1c. However, increased natural pest control were significantly associated with higher crop yields when interacted with management practices after accounting for weather, soil, and management (Table 1, Model 1c and Table 2, Model 2d). The estimate for the effect of natural pest control on crop yields is similar in magnitude to those estimated in Model 1c and similar in magnitude and significance to those reported by Dainese et al. [7] who report estimates for models using data from insecticide-free management studies only (Table 1, Model 2c). Relatedly, while the direct relationship between landscape simplicity and natural pest control is not significant, the interaction between management and landscape simplicity in Model 2f suggests that natural pest control is lower in organically managed fields where the surrounding landscape has a greater proportion of cropland (in comparison to conventional management) (Table 2, Model 2f). The net impact of this intersectional effect is, however, significant only at a 90% confidence level and is likely to be observed only for extremely high values of landscape simplicity (Fig 3). These results differ from the results of Model 1e and with the results of Dainese et al. [7] which suggest that landscape simplicity does not have a significant effect on predator activity. Like the other models, temperature, precipitation, and soil variables did not have significant direct effects on crop yield or natural pest control in Models 2d or 2f.

## Discussion

Abundance and richness of ecosystem services providers and landscape simplicity serve as critical determinants of crop yields [15, 48]. However, there exists limited knowledge on the relationships between landscape simplicity, ecosystem services, weather and soil conditions, agricultural management, and crop yields. Given the reliance of the state-of-the-knowledge on a few high-profile analyses, an understanding of how analytical modeling approach may alter or influence understanding and interpretation of findings about these relationships is warranted. Reanalysis of existing published data allows an opportunity to test the robustness of findings and may uncover new relationships that may have been overlooked or undetected in prior analyses, providing both greater nuance in understanding and greater confidence in findings [31].

In this study we conduct a reanalysis by applying different analysis methods to the published dataset from the Dainese et al. [7] meta-analysis of agricultural field experiments investigating the relationships between pollinator and predator populations, ecosystem services, and crop yields using Bayesian path analysis. Our analysis employs simple, frequentist, statistical inference techniques and focuses on the relationships between landscape simplicity and ecosystem services, landscape simplicity and yields, and ecosystem services and yields. In addition, our analyses test assumptions about the influence of weather, soil, and management through the application of explicit controls and interactive effects. Overall, the results of our analyses support the main findings of Dainese et al. [7] that suggest that greater levels of landscape simplicity are associated with lower crop yields, and that this association is in-part due to reduction of support for pollinator and predator provided ecosystem services that support yields. However, our analyses also offer an alternative perspective on the total effect of landscape simplicity on crop yields relative to the contribution of pollination and pest control to yields, and on the influence of management and soil health on the relationships between landscape simplicity and ecosystem services as well as yields.

The linear models with interactive effects and explicit controls suggest that both pollination and natural pest control have positive significant effects on crop yields after accounting for weather, soil, and management. These findings agree with the findings of Dainese et al. [7] and the magnitude and significance level of the estimated effects are commensurate. In contrast with our model without controls and the findings of Dainese et al. [7], landscape simplicity did not have a significant effect on pollination in the models with controls. Instead, our findings suggest that management context, and specifically the inclusion of seminatural habitat on farms, plays a significant role in the level of pollination services. This suggests that landscape function plays a stronger role than landscape diversity in supporting pollination services. Relatedly, our models with controls suggest that landscape simplicity does not have a significant direct effect on pest control but that this effect varies across conventional and organic management contexts. The implication of this interactive effect is that pest control in organic management contexts may be significantly adversely impacted by high levels of landscape simplicity, while in conventional management contexts the effects of landscape simplicity do not significantly alter pest control services due to compensatory effects of insecticide use. These results contrast with the results of Dainese et al. [7] and Karp et al. [16] who found weak or inconsistent effects of landscape simplicity and composition on pest control but appear to be consistent with the findings of Smith et al. [17] that biotic abundance and richness are greater in organic systems than in conventional systems.

In our linear models without interactive effects, we found that pollination has positive significant effects on crop yields. This result is consistent with Dainese et al. [7, 46] and Kennedy et al. [7, 46] who found that richness and abundance of pollinators have positive effects on crop yields. However, we did not observe a significant relationship between natural pest control, landscape simplicity, and crop yields when using all data and not explicitly controlling for management. This finding confirms the findings of Dainese et al. [7] who did not find a significant effect of pest control on yields when not accounting for insecticide use and Karp et al. [16] who used similar data and found inconsistent effects of landscape composition on pest control and yields in their models that did not explicitly account for farm management or insecticide use.

While landscape simplicity did not have significant direct effects on crop yields in our models without controls, the addition of weather, soil, and management variables in the models with controls and interactions paint a more nuanced picture. Specifically, on average, increased landscape simplicity was significantly associated with lower crop yields after accounting for soil type, but the effect of landscape simplicity on yields varied significantly across soil type. Sand and clay soils were associated with an increased negative effect of landscape simplicity on yields, while loamy soil types were associated with either no effect of landscape simplicity or a positive effect of landscape simplicity on yields.

We suggest two potential explanations for this finding. First, yields in locations with more optimal soil conditions for crop yields are likely to experience smaller relative benefits from increasing landscape simplicity due to already elevated levels of yields in comparison to locations with poorer soils and lower yields, where even slight increases may have a significant effect on total production [29]. Second, the physical, chemical, and biological properties of soils can modify plant health and vigor, impacting the susceptibility of plants and associated crop yields to biotic stressors such as pests [41]. These findings are consistent with earlier studies that biophysical soil characteristics regulate agroecosystems and related ecosystem services, with potential effects on crop yields [11, 25, 29, 49].

The total effect of landscape simplicity on crop yields suggested in the model with soil interactions is also notably larger (more than order of magnitude) than the total effect inferred from Dainese et al.’s [7] path analyses, which may indicate that mechanisms other than the provisioning of pollination and pest control services such as improved soil health resulting in reduction in plant susceptibility to stressors, are responsible for the majority of the observed effect of landscape simplicity on yields. Interestingly, models with management had no significant direct or indirect effects on yields, suggesting that the observed effect of soils on the relationships between landscape simplicity and yields is not due to differences in management across different soil types and that the relationship between landscape simplicity and yields is inconsistent within management types. This finding contrasts with the finding of Smith et al. [17] who found that landscape context influenced yields and that differences in productivity varied strongly across conventional and organic management contexts.

While climate extremes, including annual maximum temperature, have been found to have significant negative associations with crop yields [26, 27] we did not observe any significant effects of climate variables in our models. This is likely related to the relatively small size of the dataset, low variability across time, and inconsistencies in response to climate conditions across crops. In addition, we note that all our findings are strongly limited by the available data and precision of location information provided in the public version of the Dainese et al. [7] dataset. For example, our soil type measure is computed for the region in which an experiment took place, not for the specific experiment site. The climate variables are, similarly, a regional metric, that uses calendar year, rather than growing season, temperature and precipitation measures due to the large variety of crops in the dataset and varying growing seasons for each crop across experiment sites. This generalization of these control variables introduces uncertainty in their effects as well as potentially reducing their significance due to averaging of heterogeneity within regions.

Relatedly, while the published data from Dainese et al. [7] includes a crop management indicator, specific details on these management categories (such as insecticide use) is not provided. Moreover, the use of LAND (calculated as percent of cropland within a 1 km radius around the center of each field) may not consider equally important contextual factors such as landscape complexity in terms of mixed-cropping or intercropping, the presence of natural habitats at field edges, or the diversity of the broader region in which the experimental site is embedded. In addition, while many variable effects emerged as significant at a 95% or higher confidence level, in all models, regression coefficient values remained below 0.2, indicating that our models do not explain much of the variation in crop yields or ecosystem services in the dataset. However, the general consistency of our model results with the findings of Dainese et al. [7] lends some confidence to the credibility of our findings. Additional work applying methods such as bespoke Data Envelopment Analysis (DEA) methodology, which can be used to examine how differences in environmental context influence the rates at which landscape simplicity impacts ecosystem services and crop yields for relatively small sample sizes [21, 50, 51], and machine learning with synthetic dataset generation [52], may yield additional insights.

## Conclusions

A meta-analysis of a compiled global database of 1,475 crop experiments related to pollination and pest control ecosystem services and crop yields published by Dainese and colleagues has quickly garnered attention in the literature, with more than 540 citations since its publication in 2019. While Dainese and colleagues published a public version of their dataset, to our knowledge, no one has yet conducted a reanalysis of this data. Reanalysis, which uses the same data but different analysis techniques, is often overlooked as a tool for increasing confidence in results and uncovering new discoveries in favor of reproduction, which uses newly acquired data and the same analysis techniques [31].

Our reanalysis supports the findings of Dainese et al. [7], illustrating the robustness of findings that suggest that increasing landscape simplicity is associated with lower rates of pollination and pest control ecosystem service provisioning and lower crop yields. However, we also find that some estimated effects from Dainese et al. [7] are likely to be strongly dependent on management or biophysical context. We noticed that provisioning of pollination and pest control services likely accounts for only a small fraction of the total effect of landscape simplicity on crop yields and identify soil health as another potential mechanism that mediates the effects of landscape simplicity on yields. Our results complement previous findings [7, 16, 17] on landscape simplicity and ecosystem services and indicate that above and below ground ecosystem services are not mutually exclusive but concurrently contribute to support crop production in agriculture [29]. Our study results add new insight to previous studies, lend greater support to existing hypotheses about the benefits of landscape diversification on crop production and ecosystem health, and illustrate the value of applying different modeling approaches to the same data through reanalysis to further our understanding of complex processes for which collection of relevant data is an ongoing challenge.

## Supporting information

S1 Table The percentages of modal United States Department of Agriculture (USDA) topsoil texture classes extracted within each study region. (DOCX)

S1 Data (XLSX)
