# Supplementary material for: Effects of landscape simplicity on crop yield: A reanalysis of a global database
Source: PLoS One. 2023 Dec 14;18(12):e0289799. doi: 10.1371/journal.pone.0289799 (PMC10721009; doi:10.1371/journal.pone.0289799)
Supplement: S1 Table — (DOCX) [file pone.0289799.s001.docx]

**Supporting information**

**Data compilation**

We compiled the dataset from a global database on biodiversity and crop production published by Dainese et al. [7]. Since we are interested in assessing crop yields, only dataset3 and dataset4 of the global database met our criteria and were therefore included. The dataset3 contains pollination services and crop production while dataset4 contains natural pest enemy control and crop production. The two datasets were merged using RStudio version 1.4.1717. The datasets were merged while filtering and deleting duplicates by retaining only unique SiteID. The merged crop yield dataset retained 40 studies, 581 crop fields, 18 countries, 24 different crops, and 25 regions of crop field locations across the world.

Climate data were obtained by taking the average across all pixels within the regions using [‘exactextractr’](https://isciences.gitlab.io/exactextractr/reference/exact_extract.html) package in RStudio. The average across all pixels within a radius of the center of the region was calculated for each of these annual raster layers, where the radius was defined as one-half the square root of the region area. For the soil variable, aggregated soil texture class for crop experiment locations was extracted by taking the mode across all pixels within a radius of the center of the region to create the *Soil* metric (See S1 Table 1). The production (crop yield) dataset from Dainese et al. and the extracted (climate and soil) data were merged using RStudio while joining by the SiteID.

**S1 Table. The percentages of modal United States Department of Agriculture (USDA) topsoil texture classes extracted within each study region.**

| Region | Region Area (KM^2^) | Most frequent USDA texture class name | # of texture classes | % Region area in most frequent texture class |
| --- | --- | --- | --- | --- |
| Betuwe | 216 | Loam | 4 | 74.86 |
| California | 423970 | Loam | 6 | 44.39 |
| Ceara | 148886 | Sandy Loam | 6 | 43.56 |
| Centralsulawesi_Napuvalley | 61841 | Sandy Clay Loam | 4 | 53.41 |
| Chapada Diamantina | 1520 | Sandy Loam | 1 | 100 |
| Comarca Andina | 7550 | Loamy Sand | 4 | 50 |
| Cundinamarc | 22623 | Sandy Loam | 5 | 70.6 |
| Friuli Venezia Giulia | 7847 | Loam | 5 | 55.08 |
| Goias/Df | 3108 | Sandy Clay Loam | 3 | 50.27 |
| Haean Catchment | 64 | Loam | 1 | 100 |
| Kilimanjaro | 13250 | Sandy Clay Loam | 10 | 27.77 |
| Kodagu | 4102 | Sandy Clay Loam | 3 | 72.42 |
| Limburg/Overijssel | 2209 | Sand | 6 | 58.7 |
| Limpopo | 25754 | Sandy Loam | 7 | 54.08 |
| Loire | 800 | Loam | 4 | 77.78 |
| Lower Saxony | 47614 | Loam | 8 | 35.44 |
| Monteregie | 4576 | Loam | 5 | 57.57 |
| New York | 784 | Loam | 3 | 43.71 |
| Scania | 11303 | Sand | 3 | 59.65 |
| Soconusco Region, Southern Chiapas | 74211 | Sandy Loam | 7 | 44.19 |
| South Italy | 73224 | Loam | 4 | 65.9 |
| Vastergotland | 24000 | Sand | 3 | 78.53 |
| Upper Midwest | 169634 | Loam | 4 | 55.82 |
| Yorkshire | 15421 | Loam | 8 | 34.72 |
| Zurich | 34 | Clay | 5 | 47.05 |
